# Evaluation of health care providers’ role transition and satisfaction in hospital-at-home for chronic obstructive pulmonary disease exacerbations: a survey study

**DOI:** 10.1186/1472-6963-13-363

**Published:** 2013-09-27

**Authors:** Cecile MA Utens, Lucas MA Goossens, Onno CP van Schayck, Maureen PHM Rutten-vanMölken, Maria W Braken, Loes MGA van Eijsden, Frank WJM Smeenk

**Affiliations:** 1 Department of Respiratory Medicine, Catharina Hospital, Eindhoven, the Netherlands; 2 Department of General Practice, CAPHRI School for Public Health and Primary Care, Maastricht University, Maastricht, the Netherlands; 3 Institute for Medical Technology Assessment, Erasmus University, Rotterdam, the Netherlands; 4 Department of Staff nurses Nursing and Care, ZuidZorg, Veldhoven, the Netherland; 5 Department of Health Care Policy, Meander Group Zuid-Limburg, Heerlen, the Netherlands

**Keywords:** Hospital-based home care services, Health personnel, Community health nursing, Health care surveys, Professional roles, Job satisfaction

## Abstract

**Background:**

Hospital-at-home is an accepted alternative for usual hospital treatment for patients with a Chronic Obstructive Pulmonary Disease (COPD) exacerbation. The introduction of hospital-at-home may lead to changes in health care providers’ roles and responsibilities. To date, the impact on providers’ roles is unknown and in addition, little is known about the satisfaction and acceptance of care providers involved in hospital-at-home.

**Methods:**

Objective of this survey study was to investigate the role differentiation, role transitions and satisfaction of professional care providers (i.e. pulmonologists, residents, hospital respiratory nurses, generic and specialised community nurses and general practitioners) from 3 hospitals and 2 home care organisations, involved in a community-based hospital-at-home scheme. A combined multiple-choice and open-end questionnaire was administered in study participants.

**Results:**

Response rate was 10/17 in pulmonologists, 10/23 in residents, 9/12 in hospital respiratory nurses, 15/60 in generic community nurses, 6/10 in specialised community nurses and 25/47 in general practitioners. For between 66% and 100% of respondents the role in early discharge was clear and between 57% and 78% of respondents was satisfied with their role in early discharge. For nurses the role in early discharge was different compared to their role in usual care. 67% of generic community nurses felt they had sufficient knowledge and skills to monitor patients at home, compared to 100% of specialised community nurses. Specialised community nurses felt they should monitor patients. 60% of generic community nurses responded they should monitor patients at home. 78% of pulmonologists, 12% of general practitioners, 55% of hospital respiratory nurses and 48 of community nurses was satisfied with early discharge in general. For coordination of care 29% of community nurses had an unsatisfied response. For continuity of care this was 12% and 10% for hospital respiratory nurses and community nurses, respectively.

**Conclusion:**

A community-based early assisted discharge for COPD exacerbations is possible and well accepted from the perspective of health care providers’ involved. Satisfaction with the different aspects is good and the transfer of patients in the community while supervised by generic community nurses is possible. Attention should be paid to coordination and continuity of care, especially information transfer between providers.

## Background

Health care systems are being confronted with aging populations and an increasing prevalence of chronic illnesses, like Chronic Obstructive Pulmonary Disease (COPD) [1]. In COPD, exacerbations and hospitalisations are the main contributors to high health care costs and cause a continuous pressure on hospital beds [2,3]. As a reaction, alternative treatment schemes for hospital treatment are being developed. An alternative for hospital treatment is hospital-at-home, in which patients who would otherwise be hospitalised are being cared for at home by nurses [4-6]. Hospital-at-home is (partly) substituting hospital treatment with home for a limited period of time, which distinguishes it from long term community care. Depending on their design, hospital-at-home schemes aim at reducing length of hospital stay (so-called early discharge schemes), or avoiding hospital admission [7,8]. It has been proved that hospital-at-home has no negative effects on patient outcomes [4].

The introduction of hospital-at-home within a health care system may lead to changes in health care providers’ roles, as patients are transferred to primary care while they would otherwise remain treated in secondary care. In addition, little is known about the satisfaction and acceptance of health care providers involved in hospital-at-home, which is necessary to reach successful implementation of the scheme.

Three studies evaluated hospital-at-home from the perspective of one or more health care providers [9-11]. They reported positive results on the characteristics and operation of the schemes and on the satisfaction of the health care providers involved. In the United Kingdom approximately 44% of hospitals run a hospital-at-home scheme [5]. In the Netherlands, where the current study is situated, the development of hospital-at-home for COPD exacerbations did not come into existence and only available in a pilot or study, as in the current study. Our hospital-at-home scheme is a community-based early assisted discharge scheme, in which care at home is delivered by community nurses and only accepts patients with a COPD exacerbation. The impact of the transfer of patients on nurses and other health care providers’ roles in a community-based hospital-at-home scheme is unclear. The experience of nurses and all other health care providers involved in a community-based scheme is also unclear. Therefore, the current study has two aims:

 1. To describe the role differentiation and role transition of health care providers involved in a community-based, early assisted discharge, hospital-at-home scheme for COPD exacerbations.

 2. To evaluate health care provider satisfaction with a community-based, early assisted discharge, hospital-at-home scheme for COPD exacerbations.

## Methods

### Setting and design

The current study was part of a randomised controlled trial studying the effectiveness and cost-effectiveness of a community-based hospital-at-home scheme for COPD exacerbations. A detailed description of the hospital-at-home scheme, the study protocol and the intervention was published before [12]. Results on the effectiveness and cost-effectiveness analysis have been published previously [13,14]. In the multi-centre trial, patients admitted with an exacerbation of COPD were screened for participation to the trial by the reviewing physician according to the inclusion and exclusion criteria, of which a summary can be found in Table 1[12]. Patients had to be aged 40 or older, diagnosed with COPD (i.e. at least GOLD stage I and 10 smoking pack years), hospitalised with an exacerbation and competent to give informed consent. Patients were excluded when fulfilling one or more of the following criteria: major uncontrolled comorbidity, having mental disability, living outside the care region of the home care organisation, being unable to understand the program, having an indication for treatment on the intensive care unit or non-invasive ventilation, having active alcohol and/or drug abuse and having insufficient availability of informal care at home. Eligible patients received three days of usual hospital care and were then randomised into further usual hospital care or early assisted discharge. A summary of the randomisation criteria are displayed in Table 1. The early discharge group was transferred home on the fourth day of admission and received care at home for the consecutive four days. Home care was, in principle, delivered by generic community nurses of the local home care organisation. Community nurses specialised in respiratory diseases performed a follow-up visit that was scheduled between the 10th and 14th day of the overall treatment. At home, patients were not visited by a pulmonologist (in training) or general practitioner. The usual hospital care group continued to receive hospital care as usual. In total, 139 patients were included in the trial. Details of the baseline characteristics of participating patients are shown in Table 2.

**Table 1:** Inclusion and exclusion criteria and randomisation criteria

**Inclusion criteria (checked on day 1)**	**Exclusion criteria (checked on day 1)**
Age ≥ 40 years	Major uncontrolled co morbidity
Competent to give informed consent	Mental disability
Diagnosed with COPD at least GOLD stage I and 10pack years of smoking	Living outside care region of the home care organisation
Hospitalisation for COPD exacerbation	Inability to understand the program
	Indication for admission to intensive care unit or for non invasive ventilation
	Active alcohol and/or drug abuse
	Insufficient availability of informal care at home
**Randomisation criteria (checked on day 3)**	
Completed Informed Consent on day three of admission	
Acceptable general health:	
- Decrease physical complaints	
- Non dependency of therapies that cannot be given at home	
- Being able to visit toilet independently	
Normal or moderately increased blood sugar levels, defined as ≤15 mmol/L or ≥ 15 mmol/L but patient is capable to regulate blood sugar levels independently	
Respiratory complaints of dyspnoea, wheezing and rhonchi must have decreased in comparison with day of admission.	

**Table 2:** Baseline characteristics and treatment at admission

**Characteristic**	**Usual hospital care (N = 69)**	**Early assisted discharge (N = 70)**
Age (years)	67.8 (11.3)	68.3 (10.3)
Men (%)	38 (55.1)	48 (68.6)
Current smokers (%)	27 (39.1)	23 (32.9)
Comorbidity score†	1.68 (1.1)	1.74 (1.1)
Comorbidity score of 1 (%)	42 (60.0)	38 (54.0)
Comorbidity score > 1 (%)	27 (39.0)	32 (46.0)
*Living situation:*		
Living alone (%)	21 (30.4)	22 (31.4)
Receiving care at home before admission (%)	16 (23.2)	17 (24.3)
*Treatment at admission:*		
Long term oxygen treatment (%)	4 (5.8)	5 (7.1)
Oral steroids (%)	5 (7.2)	10 (14.3)
Course of oral steroids prior to admission (%)	34 (50.0)	35 (50.7)
Course antibiotics prior to admission (%)	31 (45.6)	32 (46.4)

† Charlson Comorbidity Index, 1 = only COPD, higher score means more comorbidities.

Community nurses were informed about the patient’s condition and care needs through a nursing discharge form including information on the treatment period in the hospital. The form was transferred home by the patient and available for examination to the visiting community nurses. Medical discharge notes were written by the responsible resident and sent by mail to the general practitioner, accompanied by a specific letter on the patient’s participation to the trial. A copy of the latter form was provided to patients to keep at home and bring it with them when visiting the general practitioners in case the letter had not been received yet or when making home visits.

Design of the current study among care providers was a survey, performed between March and June 2010. The multi-centre trial ran from November 2007 until March 2011. We chose the study period for the current survey study in order to avoid long recall periods and to be able to include as many respondents as possible. At that time 119 patients were included in the randomised controlled trial. The study was performed according to the Helsinki Declaration. Under Dutch law ethical approval or informed consent procedure is not required for studies not involving patients or studies in which people are not subjected to procedures or are required to follow rules of behaviour, and was therefore not sought and obtained for this study.

### Participants

Participants of the current study were the health care providers involved in the hospital-at-home scheme from both secondary and primary care. Only care providers who had contact with at least one patient who participated in the trial were invited to participate. From secondary care three teaching hospitals participated in the trial. Pulmonologists, residents in training for pulmonologist and respiratory nurses were included in the current survey. Pulmonologists and residents in training for pulmonologist were responsible for the medical care during admission and performed the two scheduled outpatient follow-up visits to the outpatient clinic during the follow-up period of the study. The follow up period was the 90 days after discharge from the hospital or the early discharge scheme. Respiratory nurses in the hospital performed the two scheduled outpatient follow-up during the 90-days follow-up period and were also in charge of the logistics of the trial activities (patient recruitment, patient data collection, etc.). The teaching hospitals were in the South-East region of the Netherlands, all having between 600 and 700 hospital beds. All three hospitals have a respiratory ward and cover a population of 200,000 to 250,000. From primary care, two home care organisations participated to the trial. Generic community nurses and community nurses with a speciality in respiratory diseases, as well as general practitioners, were included in the current survey. The community nurses monitored the patients’ recovery at home and provided counselling and reassurance to the patient and their primary informal caregiver. Medication compliance and inhalation techniques, adherence to breathing and coughing techniques, adherence to dietary advices and support of daily life activities, were addressed as well. General practitioners were informed about the patient’s participation in the trial, but were not officially involved in the early assisted discharge scheme during the trial period, as in most hospital-at-home schemes for COPD [6]. However, because this scheme has an integrated care character, we wanted to investigate general practitioners’ opinions on the possibility for transferring clinical responsibility during the home treatment period to general practitioners. General practitioners were therefore included in this study. The nurses of the hospital ward were involved in the care during the hospital admission, but had no additional or changed role and were therefore not included.

### Measurements and data collection

To our knowledge, there is no validated questionnaire available to evaluate providers’ opinions in hospital-at-home schemes. All previously performed studies developed their own questions or questionnaires in order to answer their research questions, which depended on the design of the hospital-at-home scheme. We followed the following procedure to develop a questionnaire that would provide answers to our research questions on role differentiation and transition and satisfaction: 1) based on previous literature we determined which topics would be important for the current study [6,9-11,15,16]; 2) we consulted a representative of each group of professionals that was involved in the scheme, and also had to perform activities in the scheme. We asked the representatives about the themes we identified and possible other themes that they felt were important within the early assisted discharge scheme. We also asked them to estimate the willingness of their group to answer questions and the maximum amount of time that the group would be willing to spent on this; 3) with the information of the first two steps we designed the final questionnaire for the different groups. There was no pilot testing for the questionnaire as we received positive feedback from the representatives. Not all themes were relevant to all providers and only questions for which the respondent would be able to provide a response were asked. We took into consideration the length of the questionnaire in relation to the expected response. We tried to balance between themes in order to limit the burden for professionals. Table 3 shows an overview of which themes were applicable to each group of providers. We identified two common themes: clarity of the role within hospital-at-home and general satisfaction with the hospital-at-home. Satisfaction can be considered as the outcome of an evaluation between expectations and perceived reality concerning certain aspects [17]. Themes that were applicable to only some of the providers were: role description, satisfaction with role of the professional in hospital-at-home, to what extent the role in hospital-at-home differed from that in usual care, clinical responsibility during home treatment in hospital-at-home, responsibility for monitoring patients at home, knowledge and skills of generic community nurses for monitoring patients at home, satisfaction with the quality of care, satisfaction with coordination of care (i.e. the organisation of the different activities in the care by and between the different providers and the patient) and the satisfaction with continuity of care (i.e. the continuity of the care when transferring from secondary care to primary care). The identified themes resulted in five different questionnaires, one for each group of health care providers. Questions were asked with multiple-choice options that were dichotomous (yes/no or A/B) or had five options varying from a very negative to a very positive response. Questions on role description, changes in roles, role differences in regard to normal role, responsibilities, knowledge and the question for general remarks were (partly) open-ended questions and required a written response. Pulmonologists, residents, hospital respiratory nurses and general practitioners were invited to complete the questionnaire by mail. Nurses of the home care organisation do not have a fixed working space and are not often in an office. Therefore they were invited by e-mail to complete an electronic questionnaire. All responses were anonymous with only a reference to the profession the respondent had. Non-responders were sent a reminder by mail or e-mail, one month after the first mailing during the study process.

**Table 3:** Questionnaire themes per group of professionals

	**Pulmonologist**	**Resident**	**Hospital respiratory nurse**	**Generic community nurse**	**Specialised community nurse**	**General practitioner**
*Roles*						
Description of role (open question)	X	X	X	X	X	
Is role within early assisted discharge clear?	X	X	X	X	X	X
Satisfaction with role within early assisted discharge?	X	X	X	X	X	
What do you want to change of your role in early assisted discharge? (open question)	X	X	X	X	X	
Is role in early discharge different compared to role in usual care?			X	X	X	
Who should have clinical responsibility during home treatment in early assisted discharge?	X	X				X
Who should monitor patients at home during early assisted discharge?			X	X	X	
Knowledge & skills of generic community nurses for monitoring of patients at home			X	X	X	
*Satisfaction with early discharge*						
General satisfaction with early assisted discharge	X	X	X	X	X	X
Quality of care in early assisted discharge	X	X	X	X	X	
Coordination of care in early assisted discharge			X	X	X	
Continuity of care in early assisted discharge	X	X	X	X	X	

### Analyses

Multiple choice questions were analysed with descriptive statistics, as percentage of the total number of respondents, using SPSS version 17.0, IBM. Results for the two upper and lower categories were grouped for the presentation because of the low number of responses in some groups. Open end questions were analysed per question and per groups of professionals with qualitative analysis. Two researchers (CU and LG) coded the responses independently. First round of coding was open, with no previous assumptions made on the data. In the second round of coding thematic categories were determined that represented the theme of the responses. Differences in coding were discussed until consensus was reached. If possible and applicable, responses between the groups were then compared.

## Results

Response rates varied between providers, and was respectively 10/17 (59%) in pulmonologists, 10/23 (43%) in residents, 9/12 (75%) in hospital respiratory nurses, 15/60 (25%) in generic community nurses, 6/10 (60%) in specialised community nurses and 25/47 (53%) in general practitioners.

### Roles

#### Role description

The role description of pulmonologists and resident gave themselves concerned mainly medical tasks. Most mentioned were 1) screening patients’ for eligibility for possible early discharge according to the criteria; 2) determining the medical treatment and 3) providing the patient with information on various aspects, including safety of the scheme.

Hospital respiratory nurses mainly mentioned that their tasks were study related: informing patients on the study, co-deciding on eligibility for participation together with pulmonologists and residents, and the coordination and guidance of patients during the study period.

Community nurses mentioned several activities to be part of their role in hospital-at-home. Most mentioned were 1) the observation of the patients’ symptoms, recovery and progress; 2) the care around medication intake (providing information, education, and supervision on compliance) and 3) the counselling with respect to the patients’ disease-management, like regiments and coping with the disease.

#### Role clarity

**Figure 1:**
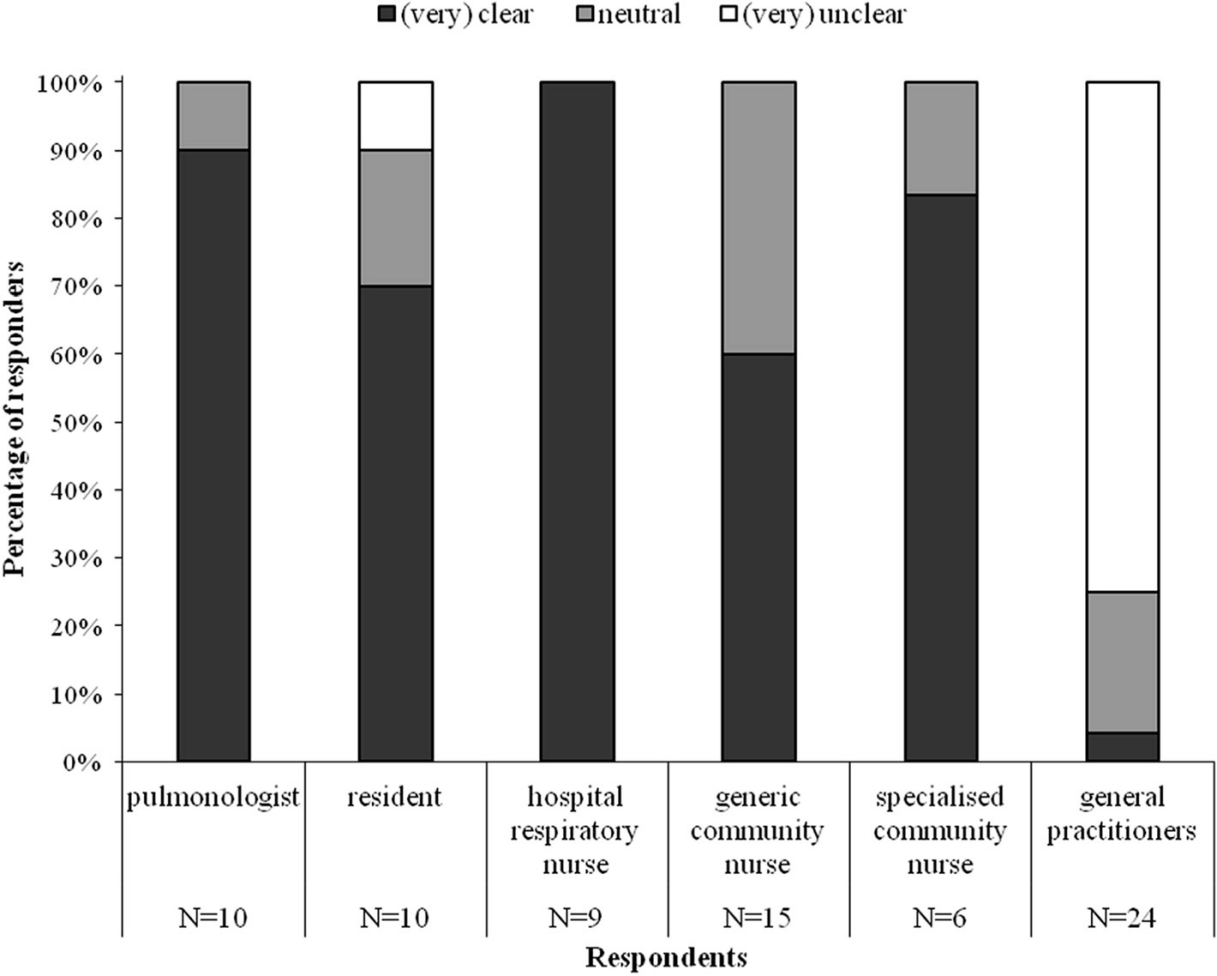
To what extend was it clear what your role as (profession) was within early discharge?

Figure 1 illustrates the role clarity among the care providers involved. For the majority of providers, except for general practitioners, their role was (very) clear.

#### Differences to usual roles and satisfaction with role in hospital-at-home

Hospital respiratory nurses and community nurses were asked whether their role in hospital-at-home was different in comparison to their usual role. Of hospital respiratory nurses, generic community nurses and specialised community nurses, 7/9 (78%), 9/15 (60%) and 4/6 (67%) believed their role was different. The differences emerging from the open responses for hospital respiratory nurses were that they now worked with patients admitted to the hospital instead of patients that come for outpatient visits, that they had to perform study related activities and had intensified contact with patients and the home care organisation. For community nurses the role had a supervising, counselling and guiding nature instead of the mainly physical role when supporting daily activities like washing and dressing.

**Figure 2:**
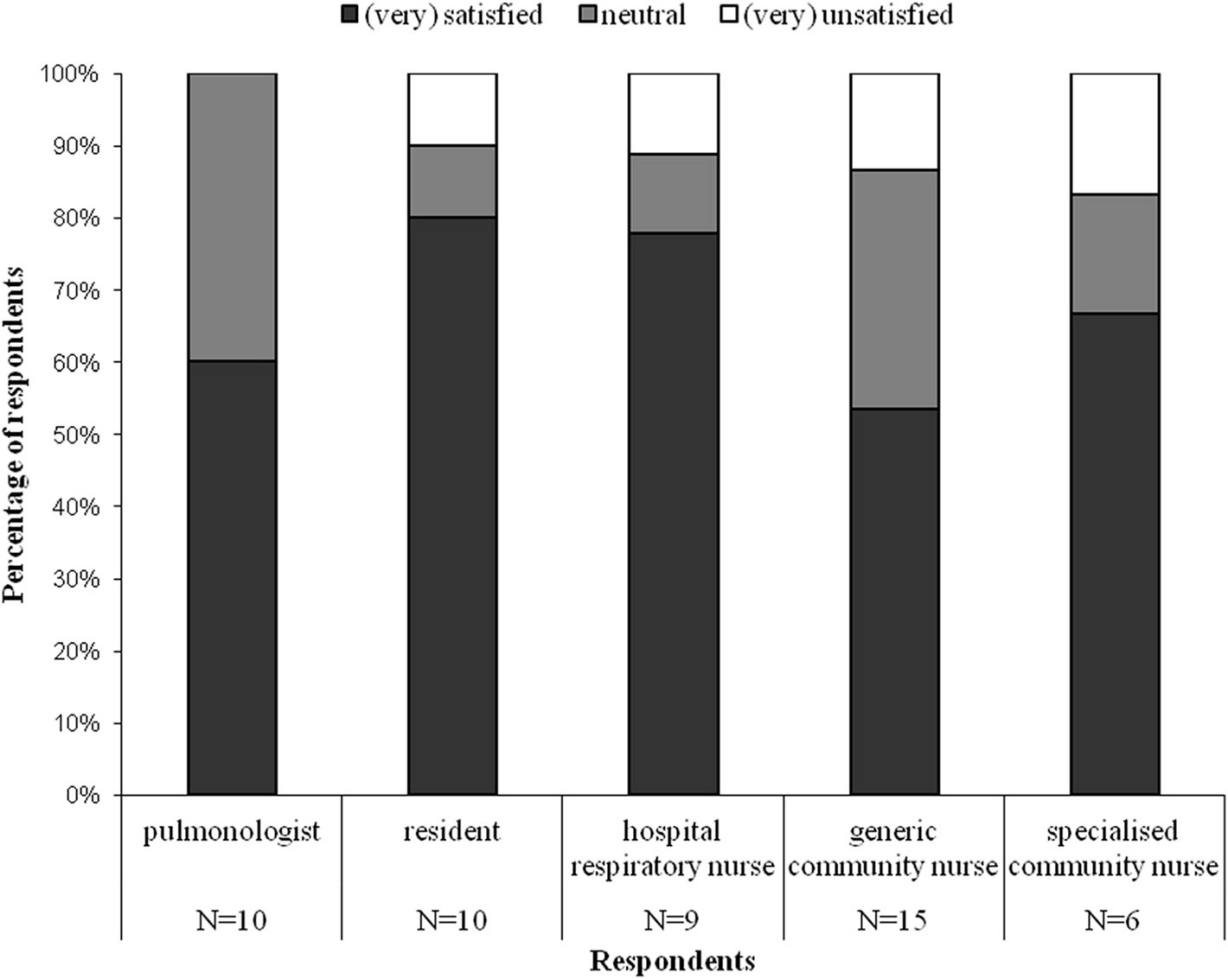
How satisfied are you with your role within early discharge?

Figure 2 shows care providers’ satisfaction with their role. Residents were more satisfied with their role than pulmonologists, and specialised community nurses were more satisfied with their role than their generic colleagues. Pulmonologists, residents and community nurses did not want to change anything about their role or were unable to make suggestions. Hospital respiratory nurses wanted to share responsibilities, as the activities for hospital-at-home came on top of their normal activities.

### Responsibilities

All specialised community nurses (6/6) and 10/15 (67%) of generic community nurses felt they had sufficient knowledge and skills to monitor patients at home.

**Figure 3:**
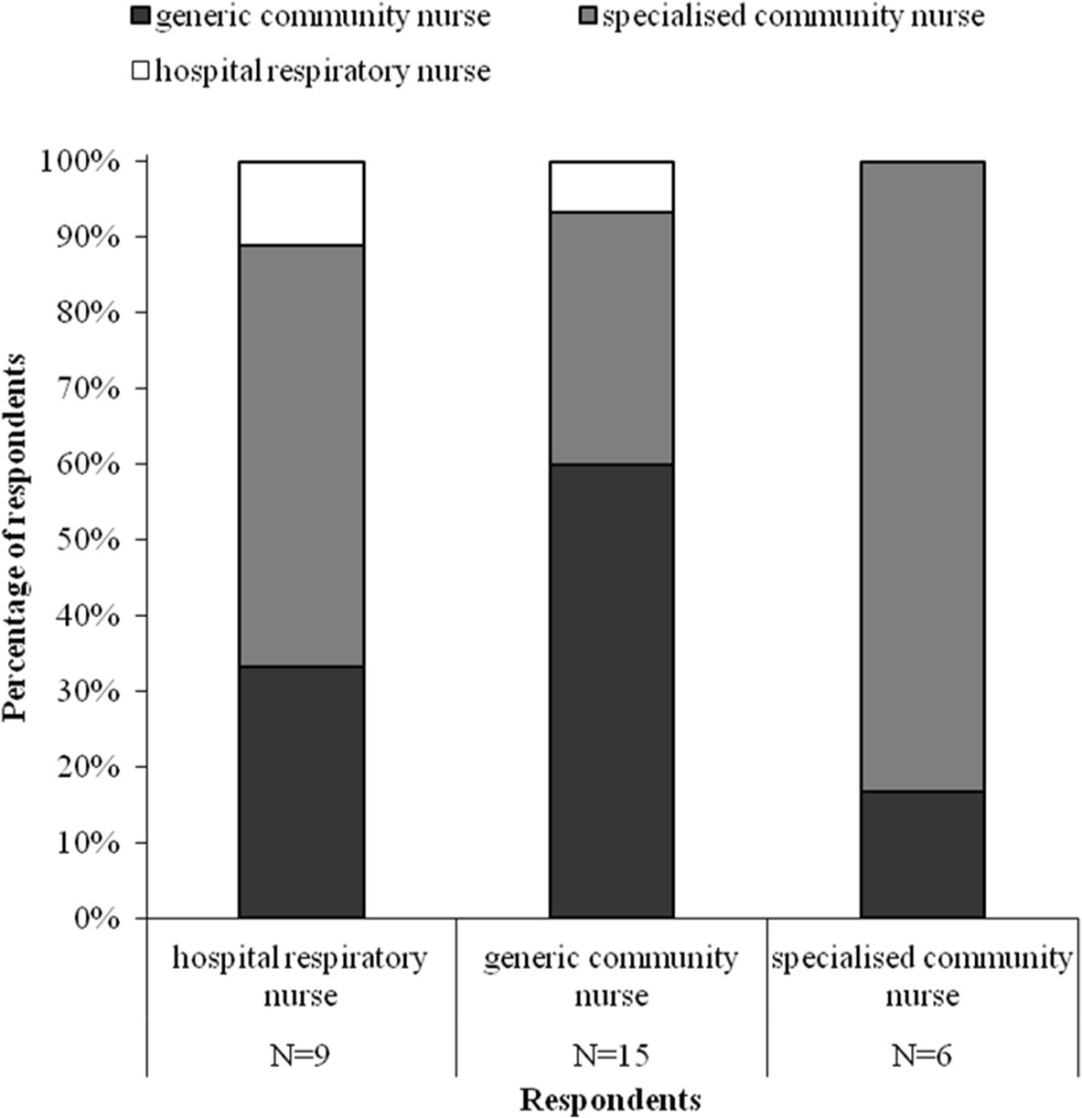
Who should monitor patients at home during early assisted discharge?

Figure 3 shows who should monitor patients at home according to the hospital and community nurses. Both generic and specialised community nurses believe they should monitor patients at home. According to 5/6 specialised community nurses, generic community nurses did have sufficient knowledge and skills to monitor patients at home. Three specialised community nurses provided additional comments, stating that generic community nurses lacked knowledge and/or skills regarding COPD, COPD patients and COPD-related medication. Two generic community nurses commented that they lacked knowledge on inhaled medication and one lacked knowledge on COPD.

**Figure 4:**
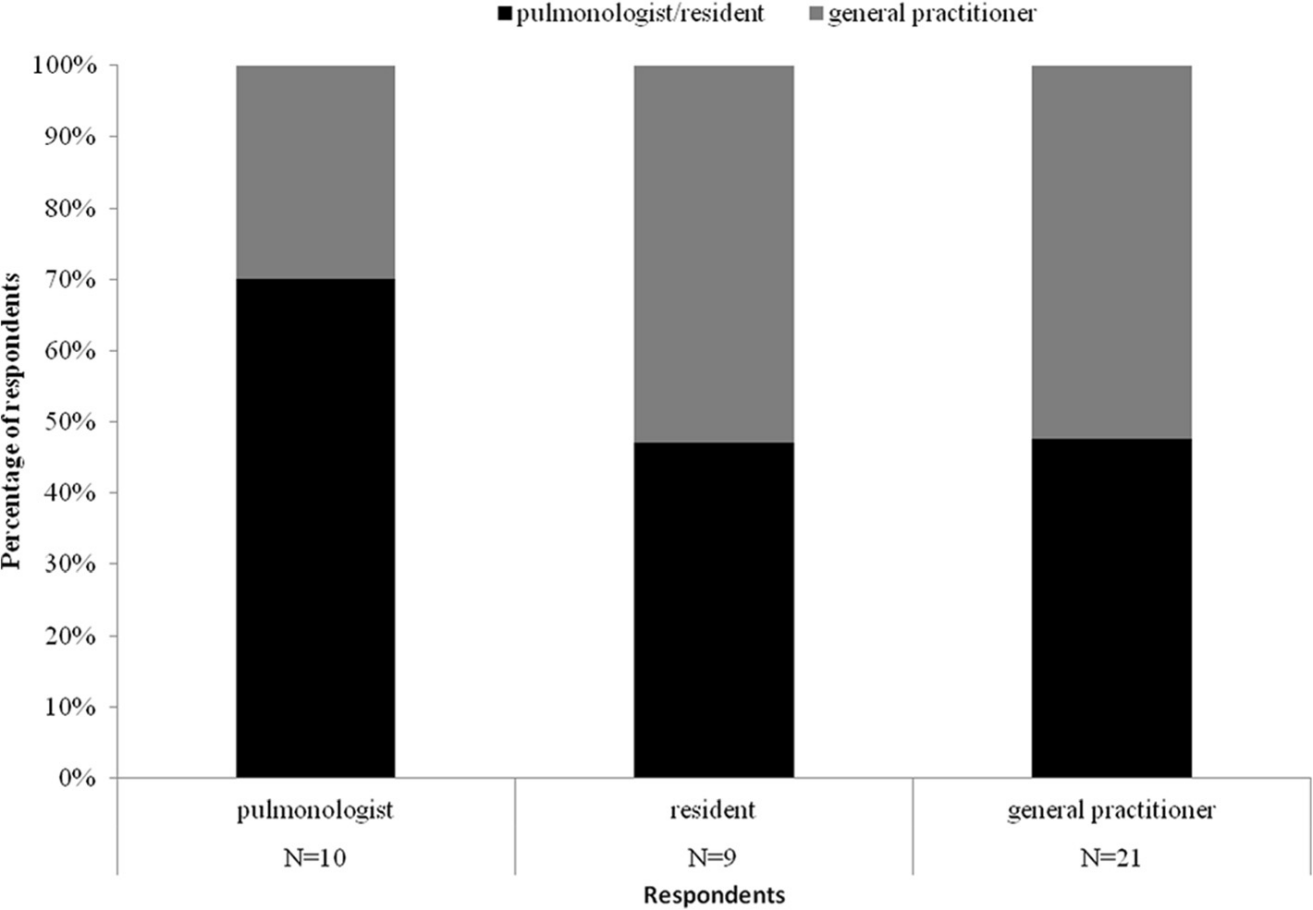
Who should have clinical responsibility during home treatment in early assisted discharge?

Figure 4 shows who should have clinical responsibility during the home treatment according to pulmonologists, residents and general practitioners. Arguments from both general practitioners and pulmonologists/residents for placing responsibility with pulmonologist are the initiation of early discharge in secondary care, presence of specific knowledge on COPD and the knowledge of course of the admission in secondary care. Arguments for general practitioner responsibility include the existing central position in primary care, easy access for patients and better sight on the general situation at home.

### Satisfaction with the aspects of the hospital-at-home scheme

**Figure 5:**
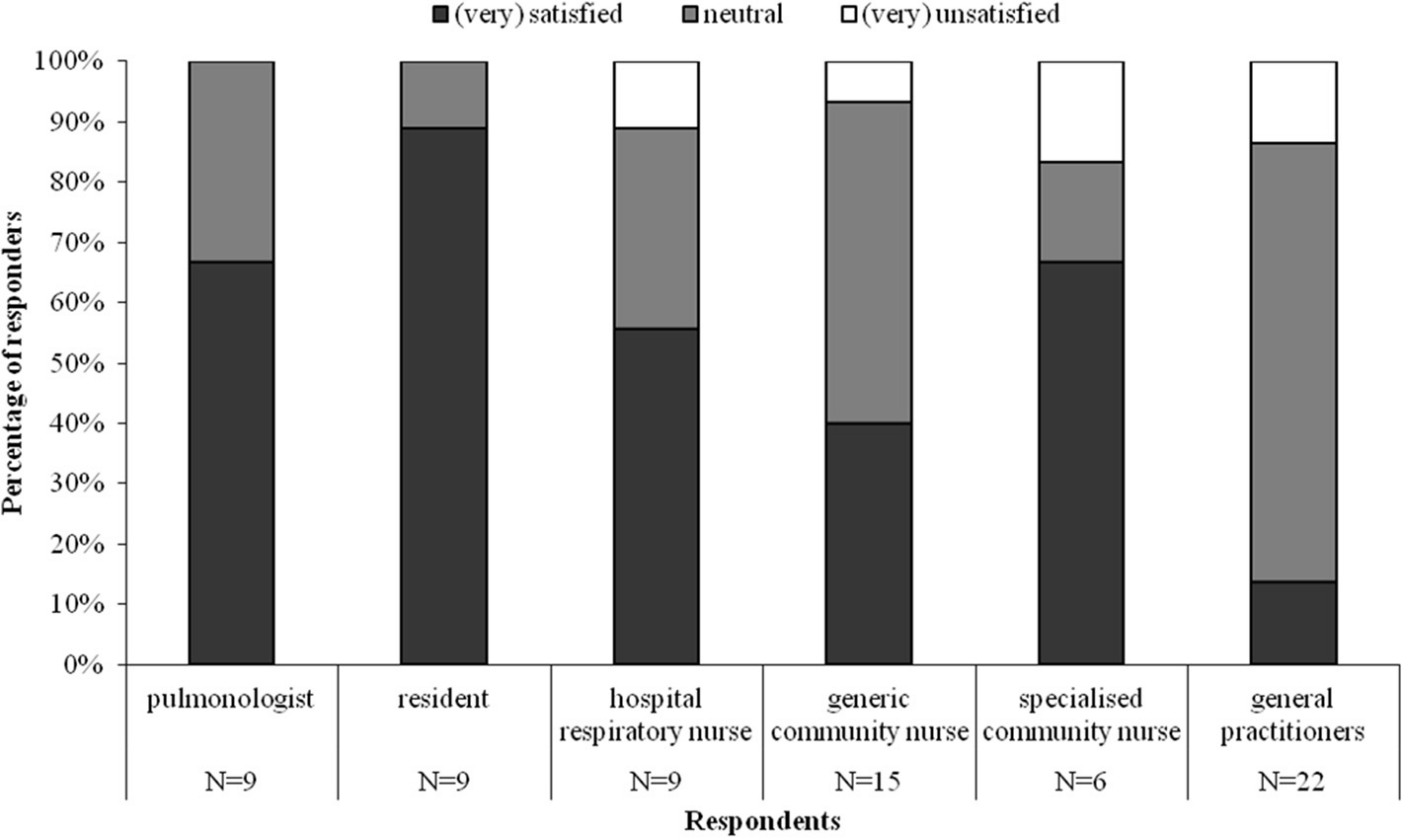
How satisfied are you with early assisted discharge in general?

**Figure 6:**
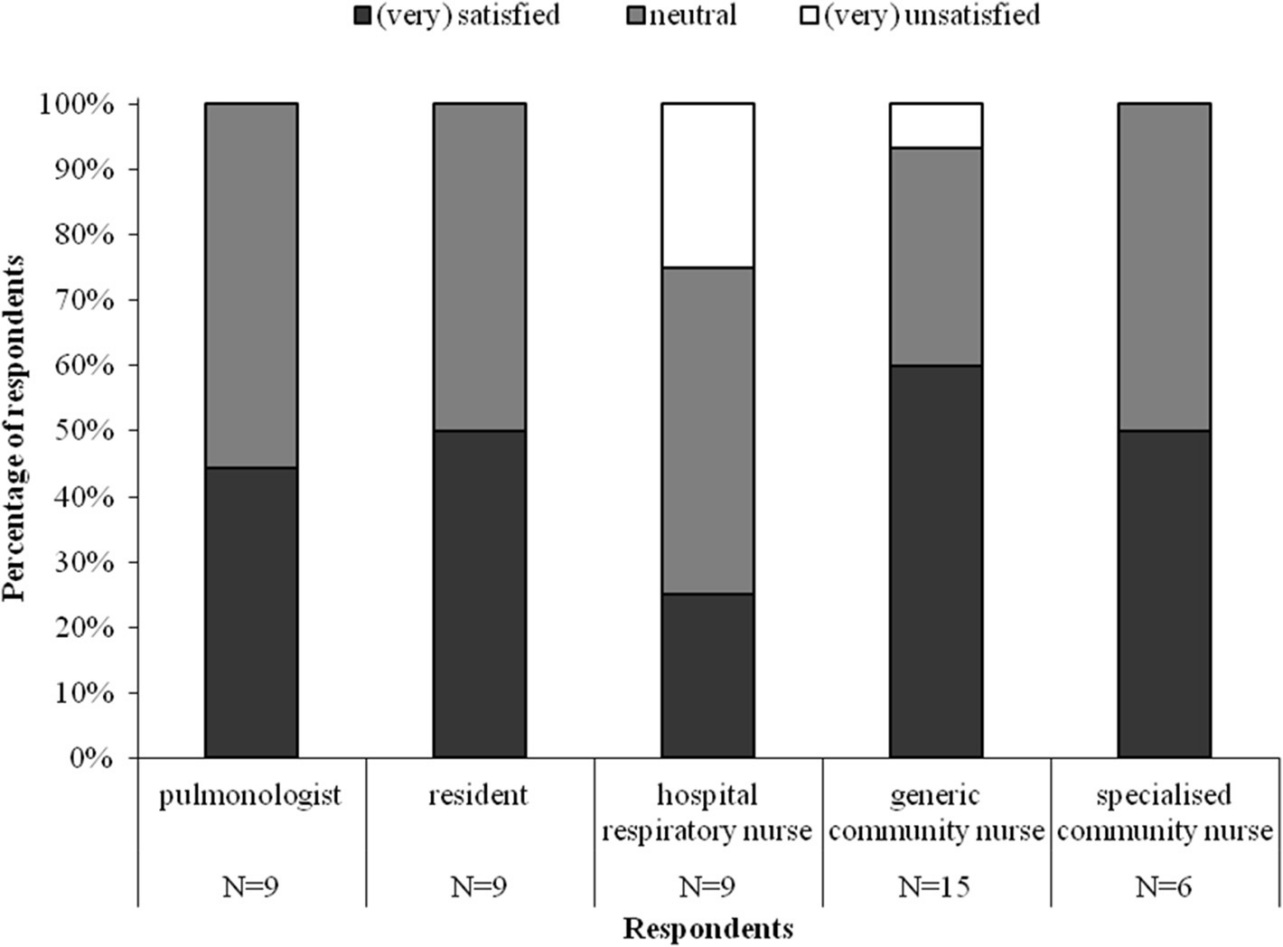
How satisfied are you with the quality of care in early discharge?

**Figure 7:**
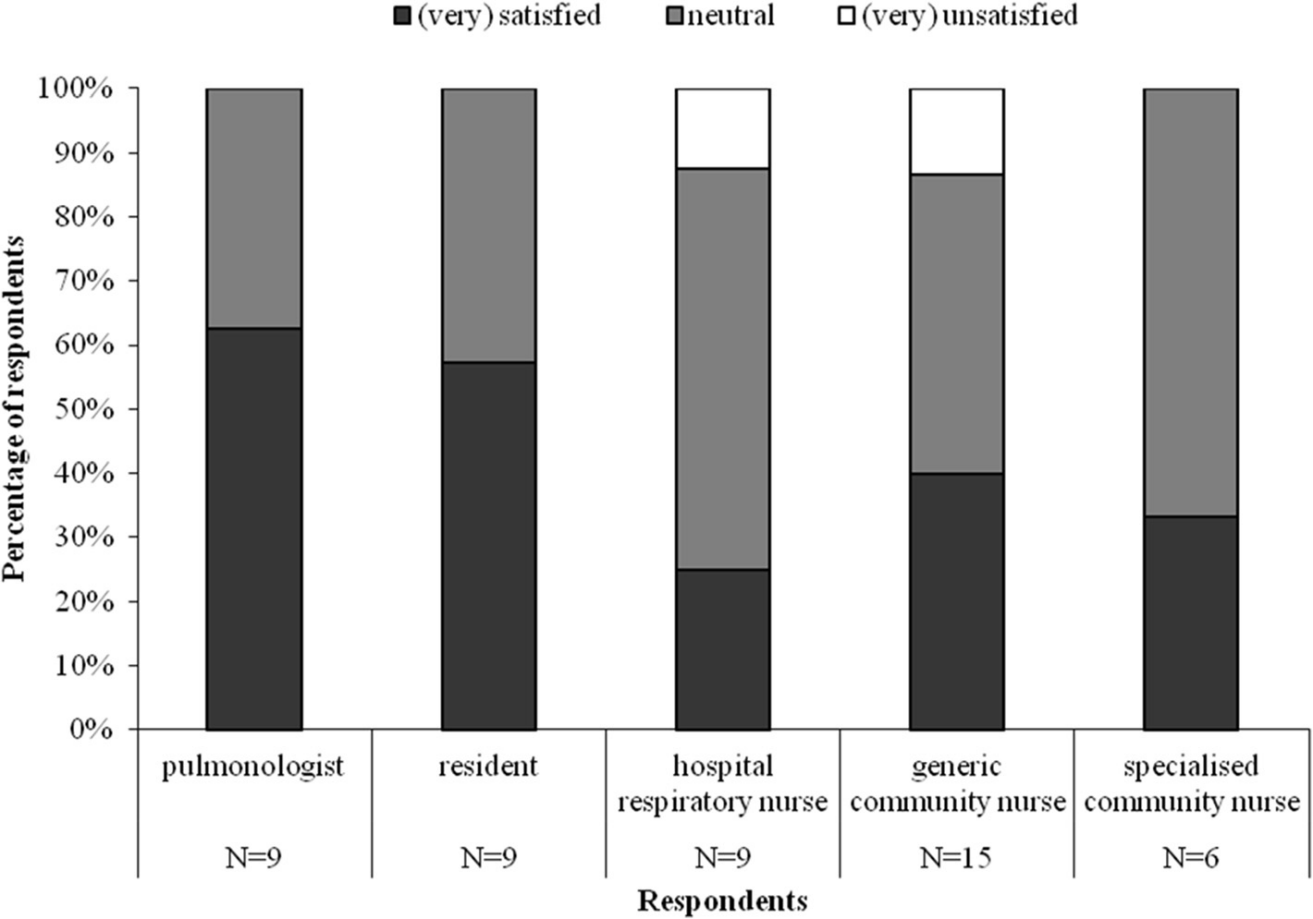
How satisfied are you with the continuity of care in early assisted discharge?

**Figure 8:**
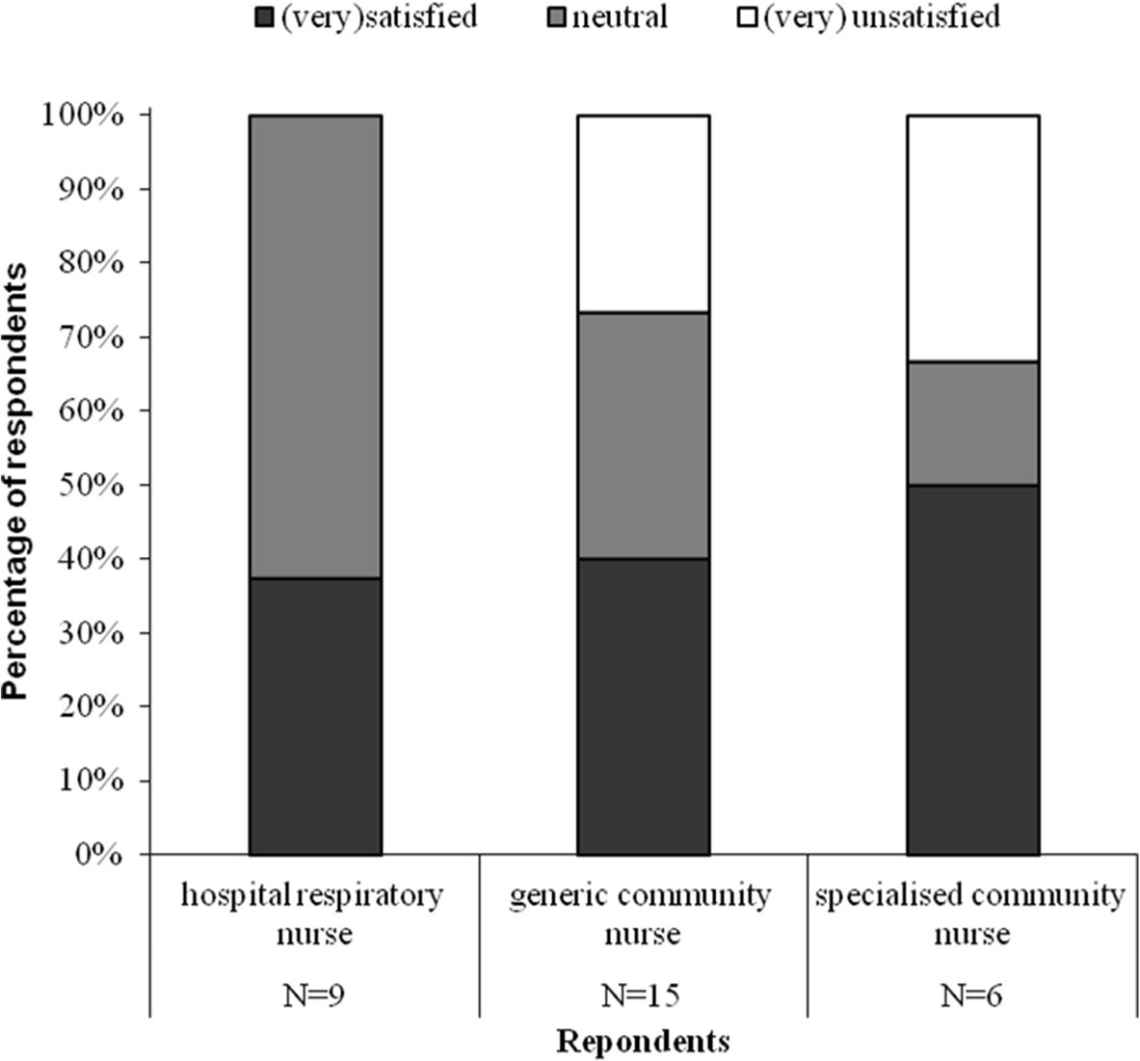
How satisfied are you with the coordination of care in early assisted discharge?

General satisfaction with hospital-at-home and satisfaction with the different aspects are displayed in Figures 5, 6, 7, 8. In general, 6/9 (67%) pulmonologists, 8/9 (89%) residents, 3/22 (14%) general practitioners, 5/9 (56%) hospital respiratory nurses, 6/15 (40%) generic community nurse and 4/6 (67%) specialised community nurses were satisfied with hospital-at-home. In generic community nurses and general practitioners 8/15 (53%) and 16/22 (72%), respectively, had a neutral response. With regard to continuity of care, the 6/9 (63%) pulmonologists and 5/9 (57%) residents was (very) satisfied, whereas 6/9 (63%) hospital respiratory nurses, 7/15 (47%) generic community nurses and 4/6 (67%) specialised community nurses had a neutral response. With regard to coordination of care, 4/15 (27%) of generic community nurses and 2/6 (33%) of specialised community nurses were not satisfied.

## Discussion

This is, to the best of our knowledge, the first study which evaluates both health care provider’s roles and satisfaction of all health care providers involved in a community-based, early discharge, hospital-at-home scheme. In general, providers evaluate the early assisted discharge scheme as positive. Coordination of care and continuity of care need attention, and the possibility for primary care providers to contact the hospital and discuss patients is valued as very important. The transfer of patients from hospital to primary care does have impact on nurses’ roles, as they described their role in hospital-at-home to be different from usual care. However, providers are satisfied with their roles and generic community nurses feel they have sufficient knowledge and skills to monitor patients at home.

The positive results on health care providers’ satisfaction with a community-based hospital-at-home scheme are confirmative to previously published results [9-11]. However, these were results on selected professionals, where our study included all professionals involved in the hospital-at-home scheme.

Except for general practitioners, who were not officially involved in the scheme, roles were clear for health care professionals. This is confirmed by the similarity to the predetermined according to the protocol and the description the professionals gave. Hospital and community nurses experienced their role to be different from usual care. Although hospital respiratory nurses reported an intensified contact with patients on the ward, differences to their roles were associated to the activities for the randomised controlled trial, whereas reported differences by community nurses were indeed associated to the patient transfer. Community nurses state that their roles changed from mainly physical activities (washing, medication dispense etc.) to more (disease specific) guiding, counselling and controlling.

Satisfaction with the different roles was high and providers did not want to make major changes. Nonetheless, there were remarkable differences between providers in primary care, who are less satisfied, and providers in secondary care. This may partly be explained by the extremely low satisfaction scores of general practitioners, but also by the changes that are opposed on primary care (i.e. community nurses). In secondary care the population treatment did not change, whereas community nurses were confronted with a ‘new’ group of patients.

The majority generic community nurses, who in practice were the professional that performed the home visits and monitored the patient, felt they had sufficient knowledge and skills for this. This contrasts with the responses of specialised community nurses. Specialised community nurses felt certain skills and knowledge lacked in generic community nurses. There seem to be different interpretations of what is necessary to monitor patients at home. As a result of more training, specialised nurses may know the specific needs of COPD patients better, and are better able to judge whether generic community nurses can monitor patients at home. However, previously performed studies on the effectiveness of early assisted discharge showed that the use of generic community nurses had no effects on patient outcomes [18,19]. In addition, another study showed that in post-rehabilitation COPD patients, delivery of home care by specialised nurses showed no superior results over care delivered by generic nurses [20]. Any deficits in the disease-specific knowledge of generic community nurses could be solved by a special education program focussing on COPD, as was done in the study of Davison et al. [18]. Due to changes in the Dutch reimbursement system, the number of specialised community nurses is decreasing. Specific and tailored training for generic nurses working with patients who are early discharge could be a good way to improve disease specific knowledge in generic community nurses and continue their work in the hospital-at-home scheme. Furthermore, specialised nurses, either working in the hospital or in the community, could be used on a consultation basis for generic community nurses. For example, when setting up a scheme it could be arranged that for each patient there is contact between the generic community nurse and a specialised nurse after the first or second home visit. In this arrangement generic community nurses receive coaching on the job, which can be reduced gradually once the scheme is running for a longer period.

It can be debated, also from a legal point of view, who should have clinical responsibility for patients that are discharged early from hospital, but still receive treatment that substitutes the hospital admission within the hospital-at-home scheme. Arguments to hold either general practitioner or hospital doctor responsible were similar among general practitioners and hospital doctors, but pulmonologist were more likely to hold responsibility at the hospital. It is possible that not only medical and safety arguments are the foundation of this opinion, but that financial issues are of importance as well [21]. However, it can be concluded that in the future it is possible that general practitioners have clinical responsibility during the treatment at home. However, although patients appreciate practitioners’ involvement after hospital stay [22], it can be debated whether this is advisable. Early discharge in our scheme was possible for a limited percentage of patients (25-30%) [14] and it would have required large involvement of general practitioners to cover the care for these patients while being treated at home. Most general practitioners from the region did not have had any patients in the schemes and those who had patients in scheme had on average 1–2 patients during the study period of 3.5 years. A shared-care model, which is the most described hospital-at-home model in the United Kingdom [5], with possibilities for fast and direct consultation of pulmonologists, could be a satisfying model for both physicians and patients. The exact design of shared-care would depend on regional arrangements between general practitioners and pulmonologists.

There were clear differences between community nurses and hospital nurses in the valuation of coordination and continuity of care. Hellesø and Fagermoen found that cultural differences between hospital and community nurses may affect coordination and continuity of care [23]. Cultural differences may influence the assessment of patients’ care needs or affect beliefs on which information is important when transferring patients from hospital to home. In addition, insufficient existing information transfer systems (i.e. transfer forms) often cause insufficient coordination and continuity of care [23]. In our study, several community nurses made comments on the inadequate information transfer and the content of the information. This may explain the different responses of community nurses and hospital nurses. A convenient, mutual designed (electronic) transfer form that covers all aspects, or an electronic patient file accessible for all professionals involved could improve coordination and continuity of care.

As in any study, there are limitations Firstly, the low response rate in some groups of professionals jeopardises the precision and generalisability of the results. In surveys there is no scientific agreement on what is considered to be the minimal response rate, but in general a response rate of 60% is considered to be the threshold for an acceptable response rate [24]. Several methods to reduce nonresponse have been applied. The length of the questionnaire has been discussed with representatives of the groups of professionals in order to minimise the burden of completing the survey. In addition, we tailored the methods for invitation and completion to the groups of professionals. Despite an electronic questionnaire to facilitate easy and fast answering and reminders to complete the questionnaire, the response rate among community nurses and residents was lower than in other groups. There are no details available on characteristics of both responders and non-responders, which complicates the assessment of reason for nonresponse and the implications of the nonresponse [25]. Possible reasons for nonresponse could be a negative attitude towards hospital-at-home. However, answers to the survey were diverse and discerning, we therefore believe that responders were representative of the study population. The larger the number of individual professionals in a group (e.g. in residents, generic community nurses and general practitioners), the lower the number of patients that the individual professionals have had contact with. This because of the distribution of patients over a larger number of professionals and, in case of community nurses, a larger working area. This may explain the lower response rates in these groups. In addition, in some cases there was a long recall period between the actual care for the early discharged patient(s) and the assessment of the questionnaire which may have some professionals decide not to fill in the questionnaire. Secondly, the measurements we used in our survey were not validated. Furthermore, it is possible that other important aspects were not addresses. For example feeling safe treating patients at home. However, other results from the effectiveness evaluation [14], patient evaluation [26] and informal caregiver evaluation [Utens et al., Informal caregiver strain, preference and satisfaction in hospital-at-home and usual hospital care for COPD exacerbations: results of a randomised controlled trial. Submitted] are positive and suggest that this probably will not be a large issue. Thirdly, (health care provider) satisfaction is a multidimensional construct that is difficult to measure. There is no gold standard for measuring this and, as we already stated, no questionnaire available to evaluate providers’ opinions on hospital-at-home schemes. Therefore, results should be interpreted with some caution, because of possible measurement error. Nonetheless, we believe that the inclusion of health care providers in the development of the questionnaire has led to an acceptable survey, representing issues that are of importance to the health care providers. Fourthly, although we evaluated important aspects of early assisted discharge, results cannot just be transferred to other countries or schemes. Transferability depends on the design of the schemes, the providers involved and the culture in organisations and among providers. More specific research should be done on more detailed aspects of hospital-at-home schemes. This could help to improve the design of the hospital-at-home schemes and to ensure care activities are executed by the most suitable professional. This creates the platform that is necessary for implementation of community-based early assisted discharge schemes.

Whether or not new health care programs should be implemented or not depends on several factors. The health care program needs to provide at least the same outcomes as usual care and from a societal perspective, cost should not be larger than in the usual care program. Furthermore, how patients and their informal caregivers evaluate the health care program is of importance. Finally, successful operation of the program depends on the acceptance of health care providers involved. In addition to positive evaluations from the perspective of patients, society and informal caregivers, this study suggests that a community-based hospital-at-home scheme is acceptable.

## Conclusions

Our findings show for the first time that the transfer of patients and treating them at home within a community-based, early assisted discharge, hospital-at-home scheme is possible from the providers’ perspective and accepted by health care professionals involved. The results from the health care provider perspective complement the evaluations on effectiveness, patient satisfaction and informal caregiver satisfaction. When implementing hospital-at-home with supervision by community nurses, attention should be to the coordination and continuity of care, information transfer and education of providers involved.

## Competing interests

The authors declare they have no competing interests.

## Authors’ contributions

CU designed the study, performed data collection, data-analysis and wrote the manuscript. LG performed data-analysis and contributed to the writing of the manuscript. OvS contributed to the design of the study and the writing of the manuscript. MB and LvE contributed to the data collection and the writing of the manuscript. MR and FS contributed to the design of the study and the writing of the manuscript. All authors read and approved the final manuscript.

## Pre-publication history

The pre-publication history for this paper can be accessed here:

http://www.biomedcentral.com/1472-6963/13/363/prepub
